# Using Structural Equation Modelling in Routine Clinical Data on Diabetes and Depression: Observational Cohort Study

**DOI:** 10.2196/22912

**Published:** 2022-04-27

**Authors:** Amy Ronaldson, Mark Freestone, Haoyuan Zhang, William Marsh, Kamaldeep Bhui

**Affiliations:** 1 Wolfson Institute of Population Health Queen Mary University of London London United Kingdom; 2 School for Electronic Engineering and Computer Science Queen Mary University of London London United Kingdom; 3 Department of Psychiatry Nuffield Department of Primary Care Sciences University of Oxford Oxford United Kingdom

**Keywords:** depression, diabetes, electronic health records, acute care, PLS-SEM, path analysis, equation modelling, accident, emergency care, emergency, structural equation modelling, clinical data

## Abstract

**Background:**

Large data sets comprising routine clinical data are becoming increasingly available for use in health research. These data sets contain many clinical variables that might not lend themselves to use in research. Structural equation modelling (SEM) is a statistical technique that might allow for the creation of “research-friendly” clinical constructs from these routine clinical variables and therefore could be an appropriate analytic method to apply more widely to routine clinical data.

**Objective:**

SEM was applied to a large data set of routine clinical data developed in East London to model well-established clinical associations. Depression is common among patients with type 2 diabetes, and is associated with poor diabetic control, increased diabetic complications, increased health service utilization, and increased health care costs. Evidence from trial data suggests that integrating psychological treatment into diabetes care can improve health status and reduce costs. Attempting to model these known associations using SEM will test the utility of this technique in routine clinical data sets.

**Methods:**

Data were cleaned extensively prior to analysis. SEM was used to investigate associations between depression, diabetic control, diabetic care, mental health treatment, and Accident & Emergency (A&E) use in patients with type 2 diabetes. The creation of the latent variables and the direction of association between latent variables in the model was based upon established clinical knowledge.

**Results:**

The results provided partial support for the application of SEM to routine clinical data. Overall, 19% (3106/16,353) of patients with type 2 diabetes had received a diagnosis of depression. In line with known clinical associations, depression was associated with worse diabetic control (β=.034, *P*<.001) and increased A&E use (β=.071, *P*<.001). However, contrary to expectation, worse diabetic control was associated with lower A&E use (β=–.055, *P*<.001) and receipt of mental health treatment did not impact upon diabetic control (*P*=.39). Receipt of diabetes care was associated with better diabetic control (β=–.072, *P*<.001), having depression (β=.018, *P*=.007), and receiving mental health treatment (β=.046, *P*<.001), which might suggest that comprehensive integrated care packages are being delivered in East London.

**Conclusions:**

Some established clinical associations were successfully modelled in a sample of patients with type 2 diabetes in a way that made clinical sense, providing partial evidence for the utility of SEM in routine clinical data. Several issues relating to data quality emerged. Data improvement would have likely enhanced the utility of SEM in this data set.

## Introduction

### Background

Currently, large amounts of routinely collected clinical data are becoming increasingly available for use in health research. The main advantages of these large-scale data sets are their comprehensive nature, and their large patient numbers [1]. Large clinical databases can improve clinical care by providing population characteristics, identifying risk factors, and allowing for the development of predictive models using vast amounts of historical data [1,2]. To date, several large data sets comprising routine clinical data have been developed in the United Kingdom and are being used to inform clinical guidance and health care delivery [3-5]. These data sets provide a rich research resource, but there are considerable limitations associated with the use of routine clinical data, particularly surrounding the completeness and accuracy of the data. Routine clinical data are subject to data entry errors, as well as systematic inconsistencies and coding errors, which can lead to inaccurate findings.

Structural equation modelling (SEM) is a statistical technique that allows for the inclusion of multiple variables and the creation of important constructs that cannot be observed directly [6]. Partial least squares SEM (PLS-SEM) is a variant of SEM that poses no distributional assumptions (eg, normality, continuous/scale) upon data used for modelling but is frequently used for predictive approaches with an aim to understanding causal structures [7]. Further, PLS-SEM can be effective with a relatively small sample: approximately 10 cases per regression or “path” estimate leading to the most connected latent variable is considered adequate, although there has been some debate about the use of PLS-SEM with very small sample sizes [7,8].

Routine clinical data contains many clinical variables that might not be directly appropriate for answering research questions. SEM could allow for the creation of clinical constructs from the routinely collected clinical variables that are more suitable for use in research. To the best of our knowledge, SEM has not yet been applied to routine clinical data. A large integrated data set has recently been developed in East London; it contains routine clinical data from both primary and secondary care [9]. This data set was developed to support commissioning decisions within health care trusts in East London, meaning that its primary purpose was not for research. Therefore, we sought to determine whether SEM could be used to make this data set more “research friendly” by attempting to create clinical constructs and model some well-known clinical associations between depression and accident & emergency (A&E) use in patients with type 2 diabetes.

### Depression, Type 2 Diabetes, and A&E Use: A Case Study

Depression has been shown to occur approximately twice as frequently in type 2 diabetes than would be predicted by chance alone [10], and is associated with increased diabetic complications and poor diabetic control [11]. Patients with comorbid depression and type 2 diabetes have been shown to have increased health care utilization [12]; for example, they are more likely to present at A&E departments [13] and have increased health care costs (up to 70%) compared to patients with type 2 diabetes without depression [14]. This is particularly marked in those with poorly controlled diabetes [15]. Successful management of depressive symptoms through the use of psychotherapy and pharmacotherapy has been found to improve diabetic control [16] and to reduce health care service use and associated costs [17,18]. The evidence cited above comes from trial data and observational studies designed specifically for research purposes. We sought to replicate these findings using large-scale routine clinical data. More specifically, we aimed to model associations between depression, diabetic care, diabetic control, and A&E utilization, while assessing the impact of current mental health care provision. We hypothesized that depression would be associated with increased diabetic complications and poor diabetic control, and that both depression and poor diabetic control would be associated with increased utilization of A&E. We predicted that the receipt of mental health treatment would improve diabetic control. We also hoped to include relevant demographic, behavioral, and clinical factors in the model that are likely associated with pathways to care for people with depression and type 2 diabetes.

## Methods

### Study Setting

We used a large patient-linked data set from the borough of Tower Hamlets, an inner-city area located in the East End of London, United Kingdom. Tower Hamlets is unique as it has a diverse population and is home to the largest Bangladeshi community in England [19]. Tower Hamlets has the highest rate of poverty, child poverty, and unemployment of any London borough [20].

### Data Source and Study Design

The patient-linked data set was developed by the Tower Hamlets Clinical Commissioning Group (CCG) and contains routinely collected clinical data from several sources: (1) Secondary Uses Service database, a secure data warehouse that stores patient-level information for management and clinical purposes other than direct patient care, and supports commissioning and the delivery of health services; (2) a primary care data set generated by North East London Commissioning Support Unit; (3) Improving Access to Psychological Therapies (IAPT) data sets (IAPT is a talking therapy service used for the treatment of adult anxiety and depression in England); and (4) clustered and nonclustered mental health care data sets (within the National Health Service [NHS], mental health care clusters provide a framework for planning and organizing mental health services and patient support).

The data set comprises data for the general practitioner–registered population in Tower Hamlets. A detailed description of the data set has been published elsewhere [9]. In this observational cohort study, routinely collected cross-sectional clinical and health service utilization data from Tower Hamlets were collated over one financial year (2017/2018). Variables of interest were selected and extracted from linked relational data sets. All data were pseudonymized and stored in a secure network database at Tower Hamlets CCG, Mile End Hospital. All data were accessed and analyzed on-site at Tower Hamlets CCG.

### Ethical Considerations

As this study was examining the utility of a statistical method, it was deemed to not be defined as research and therefore required no ethical approval. All the necessary approvals were obtained from Tower Hamlets CCG to perform the analysis on the data set.

### Participants

The sample to be analyzed included patients aged ≥18 years who were registered with a general practitioner in Tower Hamlets and had a diagnosis of type 2 diabetes recorded in their primary care records. Type 2 diabetes is deemed to be a difficult disease to reverse [21]. Therefore, all patients who ever had a type 2 diabetes diagnosis recorded were included.

### Demographic and Clinical Factors

Demographic and clinical information included age, sex, ethnicity, deprivation index, smoking status, and BMI. Information about age and sex came from primary care records. Age was treated as a continuous variable. Ethnicity was also obtained from primary care records. Patients were classified into nine ethnic groups: White, or not stated; Indian; Pakistani; Bangladeshi; other Asian; Black Caribbean; Black African; Chinese; other ethnic group. For the purposes of the analysis, patients were reclassified into two groups: White or not stated and non-White. Deprivation index was based on Census data using Lower Layer Super Output Areas. Deprivation scores ranged from 1-10, with lower deciles being indicative of higher deprivation. Information relating to BMI and smoking status came from primary care records.

### Measures of Mental Health Diagnoses and Care

Mental health variables included in the analyses were from primary care records, IAPT data, clustered mental health data sets, and nonclustered mental health data sets. Information about whether a patient had ever received a diagnosis of depression, anxiety, severe mental illness (SMI), alcohol use, or personality disorder was obtained from primary care records. The variable used for alcohol intake was generated by North East London Commissioning Support Unit. This variable contained collapsed scores for both the Alcohol Use Disorders Identification Test (AUDIT) and the AUDIT for consumption (AUDIT C) and was treated as a continuous variable in the analyses. Scores on the AUDIT range from 0-40, with higher scores indicating higher risk of dependence. The AUDIT C consists of the 3 consumption questions from the AUDIT and scores can range from 0-12, with higher scores indicating higher risk.

As the analysis was mainly concerned with depression, availing of clustered mental health care relating to depression was included in the model as well. The following NHS mental health clusters were deemed likely to be associated with depression: care cluster 1 (common mental health problems, low severity); care cluster 2 (common mental health problems, low severity with greater need); care cluster 3 (nonpsychotic, moderate severity); care cluster 4 (nonpsychotic, severe); care cluster 5 (nonpsychotic, very severe); and care cluster 15 (severe psychotic depression).

Variables that may be markers for the treatment of depression were also included in the analyses. These included whether a patient had received an antidepressant prescription from their general practitioner within that financial year, whether the patient had accessed IAPT services, and whether the patient had been admitted to a psychiatric inpatient ward. Although these variables are not necessarily specific to depression, the use of these services are increased among patients in the Tower Hamlets data set who have received depression diagnoses. Therefore, they are deemed to be an acceptable proxy for depression treatment in this case.

There was no variable relating to the use of psychiatric inpatient services readily available in the patient-linked data set. Therefore, this variable had to be constructed using information from the nonclustered mental health services data set. Within Tower Hamlets, there are six psychiatric inpatient wards: Brick Lane ward, Globe ward, Lea ward, Millharbour ward, Roman ward, and Rosebank ward. If a patient had been admitted to any of these wards within financial year 2017/2018, they were recorded as having been a psychiatric inpatient. However, the reason why the patient was admitted to a psychiatric ward was unknown.

### Measures of Diabetes Care

We included several variables relating to diabetes care and diabetic control. The diabetes care variables were taken from primary care records and comprised whether a patient had been assigned a diabetes care plan, received a diabetic retinal exam, or received a diabetic foot exam. As specified in the National Institute for Health and Care Excellence (NICE) 2019 guidelines for the treatment of type 2 diabetes in adults, when a patient receives a diagnosis of type 2 diabetes, a diabetes care plan is usually agreed between the patient and their general practitioner [1,22]. This care plan allows the patient to take responsibility for their own well-being through increasing understanding about their condition, implementing healthy lifestyle changes, and being proactive about seeking care. Receiving routine retinal and foot exams is a standard part of type 2 diabetes care used to detect any associated retinopathy or diabetic foot problems [22]. Variables pertaining to diabetic control included the patients’ latest glycated hemoglobin (HbA_1c_) levels. In this study, HbA_1c_ is measured in mmol/mol as per the International Federation of Clinical Chemistry units. HbA_1c_ is measured to determine the patient’s average blood sugar level, with higher levels being associated with more diabetic complications [23]. Both systolic blood pressure (SBP) and diastolic blood pressure (DBP) were also included as variables associated with diabetic control. Blood pressure is known to be associated with increased vascular risk in patients with type 2 diabetes and maintaining a healthy blood pressure is associated with better clinical outcomes for these patients [24].

### A&E Use

Variables used to measure A&E use related to the number of A&E attendances per patient within financial year 2017/2018 and the A&E spend associated with that patient for the same time period. This data came from the Secondary Uses Service database.

### Data Preparation and Cleaning

The data were cleaned prior to statistical analysis. In many cases, patients who had been assigned to a mental health cluster code in that year had been assigned to several cluster codes, leading to the same individual appearing in the data set numerous times. In cases where assigned cluster codes were the same, all duplicates were removed. If the assigned cluster codes were different for an individual patient, the most severe cluster code was retained, and the less severe cluster code was removed from the data set. All patients aged <18 years were removed from the data set to ensure that the analyses were being carried out on an adult sample. All variables were complete apart from AUDIT (alcohol intake) data, cholesterol data, and deprivation level. Missing AUDIT and cholesterol data were resolved using mean imputation (ie, missing values were replaced by the mean of the available cases). As less than 50 patients were missing data pertaining to deprivation level, these patients were removed from the data set. Frequency analysis revealed that there were a number of data entries well out of clinical range for HbA_1c_ values (20-100 mmol/mol), SBP (90-200 mm Hg), DBP (50-120 mm Hg), and BMI (15-55 kg/m^2^). These cases were removed from the data set.

### Structural Model

**Figure 1:**
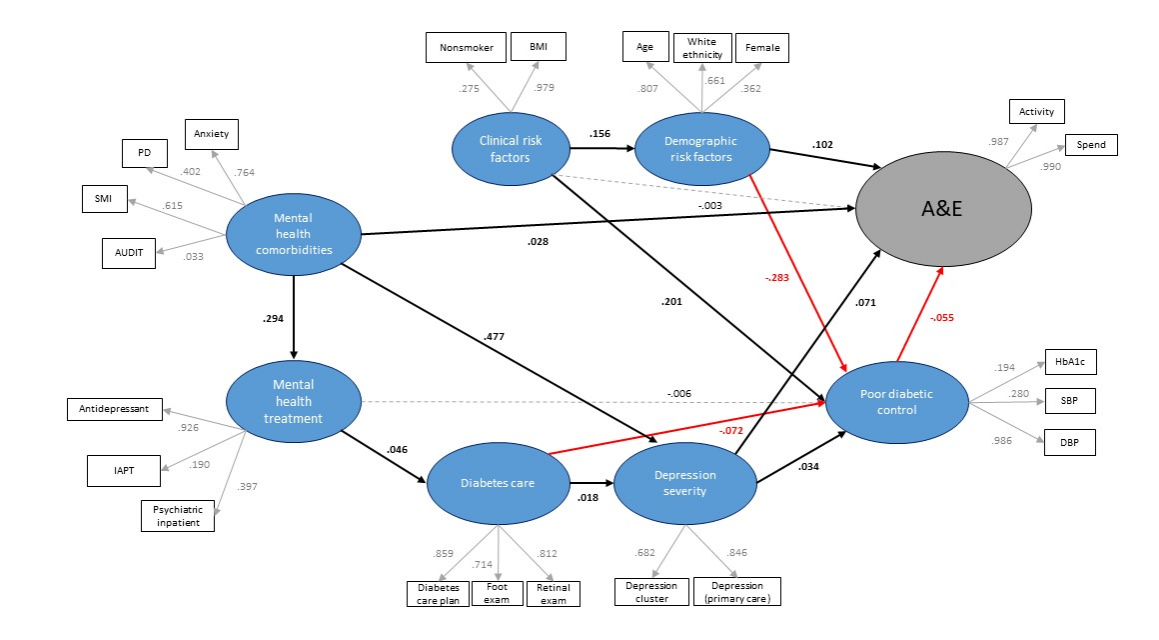
Fitted partial least squares structural equation model of factors associated with A&E use among patients with type 2 diabetes living in Tower Hamlets. A&E: Accident & Emergency; AUDIT: Alcohol Use Disorders Identification Test; DBP: diastolic blood pressure; HbA_1c_: glycated hemoglobin; IAPT: Improving Access to Psychological Therapies; PD: personality disorder; SBP: systolic blood pressure; SMI: severe mental illness.

As the purpose of this research was to test the role of mental health service use on A&E use in patients diagnosed with type 2 diabetes, we constructed a model of latent variables that reflected existing knowledge on this subject (Figure 1). Within this model, for example, we recreated the links observed between depression and poor diabetes control [11] and that the comorbidity of the two conditions increases A&E attendance [13]. We also included latent variables representing mental health comorbidity and clinical risk factors for diabetes that may confound the relationship between diabetes care, depression, and A&E admission.

### Statistical Analyses

Independent *t* tests and chi-square analyses were used to measure differences between patients with type 2 diabetes with and without depression. To investigate the relationships between depression, diabetic care, diabetic control, mental health treatment, and A&E use, PLS-SEM was carried out. Given the nature of the data, which consisted mainly of dichotomous indicators (eg, diagnoses) and ordinal measures (eg, AUDIT drinking scores) with only a small number of continuous observed variables (eg, HbA_1c_ reading), PLS-SEM was selected over other SEM approaches as it allows for the use of both continuous and discrete observed variables as indicators that measure unobservable latent variables. A covariance-based SEM approach would require continuous variables with some restrictions on distribution; Bayesian networks were also considered but are entirely probabilistic in outcome and would not have given the desired effect size coefficients for different pathways.

Our modelling approach was reflective, in that we employed observed variables from the health care data set to measure pre-existing latent variables (eg, “A&E usage”) and that, to use the typology proposed by Coltman et al [25], causality flows from latent construct to observed variable (eg, A&E usage [construct] causes increased spend on A&E services [observed]). We created 8 latent variables with multiple indicators for A&E use, poor diabetic control, diabetes care, depression severity, mental health treatment, mental health comorbidities, demographic risk factors, and clinical risk factors. PLS-SEM allowed for multiple linear equations between these 8 latent variables to be carried out simultaneously, which is not possible using traditional regression methods. The latent variables were created and connected using prior clinical and research knowledge and discussed with a clinical reference group to ensure that the proposed pathways made clinical sense.

All analyses were carried out using R software (version 3.51 for Windows x64; R Foundation for Statistical Computing) [26]; SEM analysis within R was conducted using the *plspm* package [27]. A *P* value of <.05 was considered significant.

## Results

### Patient Characteristics

Prior to data cleaning, the data set contained 20,088 patients with type 2 diabetes. Once duplicates based on mental health cluster codes were removed, the sample size was reduced to 18,092. Removal of patients under 18 years of age resulted in a sample size of 18,067 adult patients with type 2 diabetes in Tower Hamlets. Removing HbA_1c_ values (n=1382), BMI values (n=175), SBP values (n=55), and DBP values (n=55) outside of clinical range further decreased the overall sample size to 16,400. In addition, 47 patients did not have deprivation level recorded so were removed from the data set, leading to a final sample of 16,353 patients with type 2 diabetes.

Sample characteristics for the overall sample and for type 2 diabetic patients with and without depression are provided in Table 1. The overall sample comprised 7862 (48.1%) women and had a mean age of 59.5 years. The sample were on average overweight (mean BMI of 28.8) and living in areas of high deprivation (12,145/16,353, 74.3%). A considerable proportion of patients were recorded as smokers (n=4595, 28.1%), but mean AUDIT scores were low (mean 0.5), which is indicative of lower-risk drinking. In addition, 19% (n=3106) of patients with type 2 diabetes had a diagnosis of depression recorded in their primary care records, and 84.3% (n=2619) of these patients had received prescriptions for antidepressants. Very few patients with depression had been referred to local therapy services (IAPT; 1.4%) but this might reflect issues with certain data flows. Very few patients with depression had been admitted to a psychiatric ward (39/3106, 1.3%) within the study period and a greater proportion of psychiatric inpatients did not have a primary care diagnosis of depression. Overall, the majority of patients with type 2 diabetes had an agreed diabetes care plan (15,271/16,353, 93.4%) and had both a retinal (n=15,521, 94.9%) and foot (n=16,005, 97.9%) exam in the last year.

Comparisons between type 2 diabetic patients with and without depression revealed a number of significant differences in terms of demographic, clinical, and health service use factors (Table 1). Patients with and without diagnoses of depression did not differ in age but more female patients tended to have depression (*P*<.001). The majority of patients were of non-White ethnicity (12,528/16,353, 76.6%) but patients of non-White ethnicity were less likely to have a recorded diagnosis of depression (*P*<.001).

Patients with depression were more likely to be overweight (*P*<.001), more likely to smoke (*P*<.001), and scored higher on the AUDIT, indicating higher alcohol intake (*P*<.001). Patients with depression did not differ from patients without depression in terms of receiving retinal (*P=*.17) or foot (*P=*.88) exams. However, patients with type 2 diabetes and depression were more likely to have an agreed diabetes care plan (*P=*.02). Depression did not have a significant impact on HbA_1c_ levels (*P=*.46). However, patients with depression had significantly lower SBP (*P=*.004) but significantly higher DBP (*P=*.02) than patients without depression. In terms of health service utilization, patients with type 2 diabetes and depression attended A&E more in the 12-month study period than those with type 2 diabetes and no depression (*P*<.001) and incurred higher spend per head (*P*<.001). Spend, on average, for patients with type 2 diabetes with depression was £37.80 (US $49.84) more per year in A&E than for patients with type 2 diabetes without depression.

**Table 1:** Sample characteristics.

Characteristics		Overall sample (N=16,353)	Depressed (n=3106)	Not depressed (n=13,247)	*P* value^a^
Age (years), mean (SD)		59.5 (16.6)	59.5 (14.6)	59.5 (17.1)	.94
**Gender**					
	Female, n (%)	7862 (48.1)	1877 (60.4)	5985 (45.2)	<.001
	Male, n (%)	8491 (51.9)	1229 (39.6)	7262 (54.8)	N/A^b^
Non-White ethnicity, n (%)		12,528 (76.6)	1964 (63.2)	10,564 (79.7)	<.001
High deprivation^c^, n (%)		12,145 (74.3)	2297 (74)	9848 (74.4)	.30
BMI (kg/m^2^), mean (SD)		28.8 (6.2)	30.0 (6.9)	28.5 (5.9)	<.001
Smokers, n (%)		4595 (28.1)	1064 (34.3)	3531 (26.7)	<.001
Depression, n (%)		3106 (19)	N/A	N/A	N/A
Anxiety, n (%)		2498 (15.3)	1453 (46.8)	1045 (7.9)	<.001
Severe mental illness, n (%)		731 (4.5)	338 (10.9)	393 (3)	<.001
Personality disorder, n (%)		131 (0.8)	97 (3.1)	34 (0.3)	<.001
Alcohol Use Disorders Identification Test score, mean (SD)		0.5 (0.9)	0.7 (1.3)	0.5 (0.9)	<.001
Antidepressant prescribing, n (%)		7600 (46.5)	2619 (84.3)	4981 (37.6)	<.001
Improving Access to Psychological Therapies activity, n (%)		80 (0.5)	45 (1.4)	35 (0.3)	<.001
Psychiatric inpatient, n (%)		82 (0.5)	39 (1.3)	43 (0.3)	<.001
Depression cluster code^d^, n (%)		95 (0.6)	84 (2.7)	11 (0.1)	<.001
Diabetes care plan, n (%)		15,271 (93.4)	2930 (94.3)	12,341 (93.2)	.02
Retinal exam, n (%)		15,521 (94.9)	2963 (95.4)	12,558 (94.8)	.17
Foot exam, n (%)		16,005 (97.9)	3041 (97.9)	12,964 (97.9)	.88
HbA_1c_, mmol/mol (International Federation of Clinical Chemistry units), mean (SD)		57.8 (15.4)	58.0 (16.3)	57.8 (15.2)	.46
Systolic blood pressure (mm Hg), mean (SD)		127.6 (15.0)	127.0 (14.9)	127.8 (15.0)	.004
Diastolic blood pressure (mm Hg), mean (SD)		74.8 (9.6)	75.2 (9.5)	74.8 (9.6)	.02
Accident & Emergency attendances, mean (SD)		0.6 (0.9)	0.8 (1.2)	0.6 (0.9)	<.001
Accident & Emergency spend (£; US $), mean (SD)		103.80 (170.20); 136.87 (224.42)	134.50 (210.70); 177.35 (277.83)	96.70 (160); 127.51 (210.98)	<.001

^a^*P* value calculated by comparing the depressed with the nondepressed cohorts. For gender, those listed as male were compared with those listed as female.

^b^N/A: not applicable.

^c^High deprivation: combination of deciles 1 and 2.

^d^Depression cluster codes include 1, 2, 3, 4, 5, and 15.

### Structural Equation Modelling

The SEM diagram in Figure 1 depicts the relationships between the latent variables and their indicators (outer model) and the relationships among the latent variables (inner model) that make up the SEM. Latent variables are shown as ellipses and observed variables are shown as squares. Arrows show the hypothesized direction of effect between variables and each arrow is accompanied by a path coefficient, which can be interpreted as standardized beta coefficients in a regression model. Statistically significant associations between variables are shown using bold arrows. Black arrows depict positive associations whereas red arrows depict negative associations. Associations that are not statistically significant are illustrated using dashed lines.

In the final inner model, coefficients were estimated simultaneously for all 8 latent variables as depicted in Figure 1. Path coefficients are provided in Table 2 and shown in Figure 1. When checking the model, it was decided to omit deprivation index from the model as this indicator did not load on to the latent variable for demographic factors significantly.

**Table 2:** Parameter estimates from final structural equation modelling.

Parameter		Coefficient (SE)	*t* value (*df*=240)	*P* value
**Accident & Emergency on**				
	Demographic risk factors	0.102 (0.008)	12.50	<.001
	Clinical risk factors	–0.003 (0.008)	–0.448	.65
	Mental health comorbidities	0.028 (0.009)	3.18	.001
	Depression severity	0.071 (0.009)	7.97	<.001
	Poor diabetic control	–0.055 (0.008)	–6.72	<.001
**Poor diabetic control on**				
	Demographic risk factors	–0.283 (0.007)	–37.50	<.001
	Clinical risk factors	0.201 (0.007)	26.80	<.001
	Mental health treatment	–0.006 (0.008)	–0.856	.39
	Diabetes care	–0.072 (0.007)	–9.68	<.001
	Depression severity	0.034 (0.008)	4.27	<.001
**Depression severity on**				
	Mental health comorbidities	0.477 (0.007)	69.4	<.001
	Diabetes care	0.018 (0.007)	2.68	.007
Diabetes care on mental health treatment		0.046 (0.008)	5.89	<.001
Mental health treatment on mental health comorbidities		0.294 (0.007)	39.3	<.001
Clinical risk factors on demographic risk factors		0.156 (0.008)	20.2	<.001

In the final model, depression severity was associated with worse diabetic control (β=.034, *P*<.001) and higher A&E use (β=.071, *P*<.001). However, poor diabetic control was associated with lower A&E use (β=–.055, *P*<.001). Mental health treatment was not significantly associated with poor diabetic control (*P*=.39). Receipt of diabetes care was negatively associated with poor diabetic control (β=–.072, *P*<.001). Receipt of diabetes care was also associated with depression severity (β=.018, *P*=.007) and receipt of mental health treatment (β=.046, *P*<.001).

Demographic risk factors associated with A&E use (β=.102, *P*<.001) included being older, female, and of White ethnicity. These same factors were negatively associated with poor diabetic control (β=–.283, *P*<.001), meaning that being older, female, and of White ethnicity is associated with better diabetic control. Smoking and having a higher BMI were associated with worse diabetic control (β=.201, *P*<.001).

## Discussion

### Principal Findings

In this study, we sought to test whether SEM could be applied to a large routine clinical data set from East London to model known associations between depression, diabetic care, diabetic control, A&E utilization, and mental health care provision in patients with type 2 diabetes.

The model showed that depression severity was associated with worse diabetic control among patients with type 2 diabetes. This is in keeping with previous epidemiological evidence that has shown that depression is associated with increased diabetic complications and poor diabetic control [11]. Depression was associated with increased A&E utilization among patients with type 2 diabetes, which is in line with previous research [12-14]. What this suggests is that the application of SEM to this routine clinical data set enabled us to model associations in a way that made clinical sense and was in agreement with existing research. However, poor diabetic control was associated with lower A&E utilization, which is not consistent with existing evidence [15]. It is possible that this association is valid and reasons for type 2 diabetic patients with depression presenting at A&E are related to factors not associated with diabetic control. In fact, the presence of hypertension and obesity in patients with type 2 diabetes has been associated with increased A&E visits [25]. It is also possible that poor diabetic control results in greater utilization of primary care services, as well as inpatient and outpatient services. Future attempts to model associations between depression and A&E usage in type 2 diabetic patients should include relevant physical comorbidities (eg, coronary heart disease, hypertension, obesity), examine the reasons for A&E attendance, and include use of other health services in the model.

We predicted that receiving mental health treatment would be associated with improved diabetic control, thereby impacting upon health service use. However, receipt of mental health treatment was not associated with poor diabetic control in this study. This is not in agreement with previous research, which has shown that improvement of depressive symptoms through the use of psychotherapy and pharmacotherapy is associated with improved glycemic control [16]. The opposite association reported in the current study is likely related to issues with data quality, which will be outlined later. We found that better diabetic control was associated with receipt of diabetes care within primary care settings. Moreover, receiving diabetes care was also associated with depression and receipt of mental health treatment. This indicates that patients with type 2 diabetes and comorbid depression might be receiving better overall care, suggesting that comprehensive integrated care packages are being delivered in East London.

Taken together, these results provide partial support for the use of SEM in large routine clinical data sets. The data allowed us to model some associations within a sample of patients with type 2 diabetes that made clinical sense. Counterintuitive results are likely related to issues with the data set, rather than with the use of SEM. This implies that this methodology could be adapted and applied to data sets of this nature to understand pathways to health service use in other comorbid patient groups.

### Limitations

Large-scale routinely collected clinical data can have some significant limitations, particularly surrounding data completeness and accuracy [1]. In this study, the data needed to undergo considerable cleaning before analysis could take place. The removal of duplicate cases, cases where variables were way out of clinical range, and cases where data were missing and could not be imputed led to a decrease in sample size of almost 19%. These issues are mainly attributable to data entry errors and are largely unavoidable, but errors in coding and recording need improvement to support wider use of routine data in health research.

There were also suspect flaws in the data set, which may account for some of the unexpected findings we report. IAPT referrals seem suspiciously low (1.4%) in the patients with recorded diagnoses of depression. In Tower Hamlets, about 29% of patients with anxiety or depression access IAPT services [28]. This discrepancy probably reflects an issue with the flow of data. The problem with the IAPT data likely affected the mental health treatment latent variable in the SEM and might help to explain why mental health treatment was not associated with poor diabetic control.

We were unable to generate any robust goodness-of-fit statistics for the specified SEM model into the data (eg, normed fit index, standardized root mean squared residual) as these are not implemented in the *plspm* package, and data protection restrictions in place on the analysis environment meant that we could not install external software packages (eg, SmartPLS) designed to generate such statistics. The goodness-of-fit statistic generated by this package is not standardized and does not represent a “fit” measure [29]. Therefore, we could not be sure that our model was a good or a poor fit to the data; however, this was not our original intention.

A final significant limitation of this study is the cross-sectional nature of the data, meaning that causality could not be attributed in the SEM we report. Although the data we analyzed were collected over one financial year, we had no temporal information about the data, meaning that prospective analyses were not possible. This was problematic for the direction of effect we report in this study. For example, we could not tell when the latest HbA_1c_ or blood pressure measurement was taken, and we did not know the date on which A&E attendances took place. This means that the measure of diabetic control might have been taken after the A&E attendances took place within that financial year, making the attribution of causality difficult. This also might have explained the counterintuitive result seen in the SEM. Moreover, we could not tell how long a person had diabetes or depression for, which would have provided a good proxy for disease severity, and we also did not have information about how long a person had been receiving treatment for diabetes and/or depression. Despite these shortcomings, a lot of the results we report make clinical sense, supporting the application of SEM in routine clinical data. The quality of the data will determine the utility of the SEM.

### Future Directions and Recommendations

To confirm the validity of this study, it would be prudent to apply SEM to another London-based routine clinical data set in this same patient group. This would help to overcome some of the limitations outlined above and provide further evidence for utility of SEM in routine clinical data sets. Future analyses should seek to use temporal data so that prospective analysis is possible. This would allow the direction of association within the SEM to be confirmed and causality attributed to the model, overcoming some of the significant limitations outlined above. Temporal information surrounding receipt of treatment and duration of disease would also allow for the construction and inclusion of latent variables that are more clinically valid. Improvement of data flows (eg, information about use of IAPT services) and more years of data would address issues around lack of temporality and inaccurate findings.

### Conclusions

In conclusion, our results indicate that, despite the significant limitations of the data set, we were still able to successfully model associations between depression and A&E use in a sample of diabetic patients in a way that made clinical sense using SEM. This demonstrates the utility of this statistical technique in routine clinical data, and this model can be refined and retested as more data become available and prospective analyses can be carried out. Results also suggest that SEM could be adapted and applied to routine clinical data for use in other patient groups to model health care pathways.

## Abbreviations

A&E: Accident & Emergency
AUDIT: Alcohol Use Disorders Identification Test
AUDIT C: Alcohol Use Disorders Identification Test for consumption
CCG: Clinical Commissioning Group
DBP: diastolic blood pressure
HbA_1c_: glycated hemoglobin
IAPT: Improving Access to Psychological Therapies
PLS-SEM: partial least squares structural equation modelling
NHS: National Health Service
SBP: systolic blood pressure
SEM: structural equation modelling
SMI: severe mental illness
